# A Photocurable Polysaccharide-Based Hydrogel Delivery of Polydeoxyribonucleotide-Loaded Vectors for Wound Treatment

**DOI:** 10.3390/molecules28196788

**Published:** 2023-09-24

**Authors:** Zonghui Li, Xiaojun Ma, Qiang Gao, Mingxin Zhang, Hao Hu

**Affiliations:** 1 Department of Dermatology, The First Affiliated Hospital of Soochow University, Suzhou 215000, China; muliangqiuyan@126.com; 2 The Affiliated Hospital of Qingdao University, Qingdao 266071, China; maxj1985@126.com; 3 Department of Urology, Qingdao Women’s and Children’s Hospital of Qingdao University, Qingdao 266071, China; gq3@163.com; 4 Institute of Biomedical Materials and Engineering, College of Materials Science and Engineering, Qingdao University, Qingdao 266071, China

**Keywords:** polysaccharide, chitosan, sodium alginate, hydrogel, polydeoxyribonucleotide, wound treatment

## Abstract

The wounds caused by war, accidents, and diseases require timely and effective treatment. Polysaccharides, as natural macromolecules, have good biocompatibility and unique functions, and are excellent substrates for constructing new wound dressings. Short-chain chitosan (SCS) has good water solubility and, importantly, retains a large number of active amino groups. We first introduce double bonds to SCS. This chitosan derivative can be entangled with sodium alginate (SA) through electrostatic interaction. The flowing sol can be applied to a wound with an irregular shape. Under the initiation of a photoinitiator, the internal double bonds are broken and cross-linked to form a gel. The prepared hydrogel wound dressing exhibited good antibacterial properties and can provide a microenvironment conducive to wound repair. A polydeoxyribonucleotide (PDRN) has been proven to have encouraging therapeutic effects for wound healing. PDRN can be condensed by branched polyethylenimine (PEI) to form a nucleic acid delivery system, which can be efficiently uptaken by cells. The cooperation of hydrogel and nucleic-acid-based therapy presented good results in a mouse full-thickness skin wound model.

## 1. Introduction

Wound dressings are of great significance for the healing of chronic and acute wounds. Currently, commercial high-performance wound dressings could be classified into three types: membrane, hydrogel, and foam (sponge) [1,2]. Compared to old-style dressings such as bandages and gauzes, these new dressings typically have one or more of the following properties: absorbing exudation, rapid hemostasis, antibacterial, repair promotion, and easy removal [3]. Hydrogels have three-dimensional (3D) cross-linked networks that can absorb liquids thousands of times their dry weight. A microclimate conducive to wound healing can be maintained between the wound bed and the hydrogel, and the attack of pollutants such as bacteria can be prevented [4,5].

In recent years, polysaccharides have been candidates for a number of biomedical applications because of their good biocompatibility, abundant reserves, and unique biological characteristics [6]. Polysaccharide-based hydrogel has antibacterial, anti-inflammatory, hemostatic, and other characteristics, which can provide excellent wound management and accelerate wound healing. However, a single polysaccharide hydrogel may not meet the diversified needs of wound healing. Chitosan possesses plenty of amino groups on its molecular chain with a positive charge, so it has inherent antibacterial activity [7]. However, the poor water solubility of chitosan with a high molecular weight limits its widespread application [8]. The carboxymethyl modification of chitosan enhances its water solubility, but its antibacterial property will be greatly reduced [9]. Another way to improve the solubility of chitosan is to reduce the length of the molecular chain appropriately, so as to reduce the interaction force within the molecule. In this way, the amino group of chitosan can be retained and play an antibacterial role. Alginate derived from algae is also widely used in tissue engineering down to its biodegradability, non-toxicity, and non-immunogenicity [10]. Remarkably, α-L-guluronic acid (namely G-Residue) can chelate with multivalent cations (e.g., Ca^2+^ and Mg^2+^), so that adjacent molecular chains are cross-linked to form gels [11]. In an aqueous solution, sodium alginate will be negatively charged, which can generate electrostatic attraction with a polycation to form physical cross-linking. However, physical cross-linked hydrogels usually have weak mechanical strength. Chemical cross-linking between molecular chains can effectively improve mechanical properties [5].

Nucleic-acid-based therapy is to introduce the target gene into the cells and regulate the behavior of cells by expressing exogenous proteins or inhibiting the expression of specific genes [12,13]. A polydeoxyribonucleotide (PDRN) is a series of DNA fragments (50~1500 kDa) extracted from the sperm cells of Oncorhynchus mykiss or Oncorhynchus keta. PDRN has been proven to have encouraging therapeutic effects, such as promoting angiogenesis, improving cell activity, increasing collagen synthesis, and producing anti-inflammatory reactions [14]. However, how to efficiently deliver PDRN to the cells of a wound is a challenge. In the process of nucleic acid delivery to cells, nucleic acids are easy to degrade, so a vector that can effectively deliver nucleic acids to target cells is needed. Branched polyethylenimine (PEI, 25 kDa) as a common non-viral gene vector has the advantages of easy preparation and high transfection efficiency, so it is called a “gold standard” [15]. It is of great significance to deliver PDRN to the cells at the wound site using nano-nucleic-acid-delivery systems.

**Figure 1:**
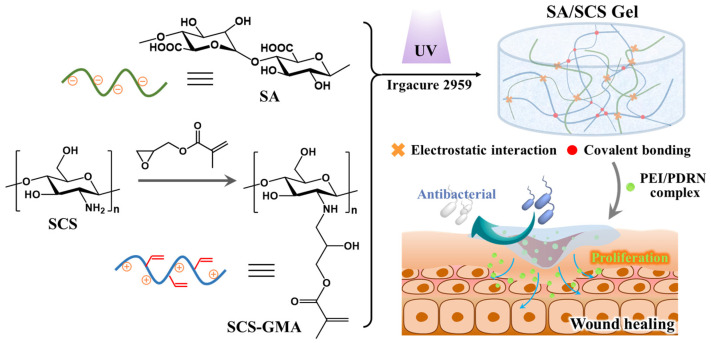
Illustration of the preparation process and wound treatment of the PDRN-vector-loaded polysaccharide-based hydrogel.

In this study, we focus on low-molecular-weight chitosan (short-chain chitosan (SCS)), which has a good water solubility and antibacterial property. As shown in Figure 1, SCS was modified using glycidyl methacrylate (GMA) to introduce double bonds to the SCS skeleton (termed as SCS-GMA) while retaining a large number of amino groups to maintain its antibacterial property. When sodium alginate (SA) is mixed with modified chitosan, positively charged SCS-GMA and negatively charged SA are entangled through the electrostatic interaction. The subsequent addition of a UV initiator will trigger an addition reaction between the double bonds, allowing the SCS-GMA to be linked through covalent bonds to form a network structure. This uniform double network hydrogel not only has good water absorption and retention performance but also has good mechanical properties. The fluidity before photo-cross-linking allows the dressing to be applied to irregularly shaped wounds [16]. In addition, photocurable hydrogels are of great significance for novel applications such as brain–machine interfaces and biosensors [17,18]. The chitosan component makes the dressing have antibacterial activity. PEI was complexed with PDRN to form nanoscale complexes and adsorbed in the hydrogel. PDRN was protected before being delivered to the wound safely. The treatment effect of the PDRN-loaded hydrogel was evaluated with a mouse full-thickness skin wound model. Satisfactory healing was observed after 2 weeks of treatment. The proposed polysaccharide-based hydrogel is a wound dressing with excellent performance.

## 2. Results and Discussion

### 2.1. Preparation and Characterization of the SCS-GMA 

**Figure 2:**
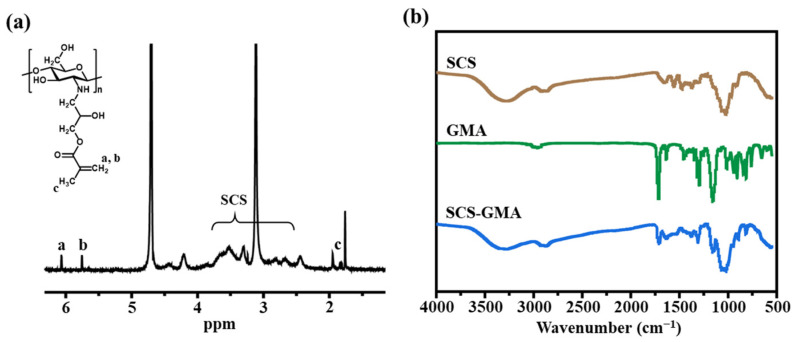
The (**a**) ^1^H NMR spectrum of SCS-GMA and (**b**) FTIR spectra of SCS, GMA, and SCS-GMA.

The SCS possesses better water solubility than the chitosan with a high molecular weight, and abundant active amino groups remained on the skeleton. The good water solubility enables the reaction to proceed under neutral conditions and ensures the uniformity of the structure and composition of the prepared hydrogel. GMA molecules contain both active vinyl and epoxy groups. The presence of two functional groups makes GMA a generalist. The epoxy group in GMA reacts with the amino group, thus introducing a certain number of double bonds onto the chitosan skeleton. The introduction of double bonds provides sites for the subsequent cross-linking of hydrogels. As revealed in the ^1^H NMR spectrum (Figure 2a), the protonation degree shifts of -CH_3_ (1.89 ppm) and the two protonation degree shifts of C=C (5.72 ppm and 6.12 ppm) verified the successful preparation of SCS-GMA. The substitution degree of the amino group in the molecular chain is about 9.5%, which was calculated with the integral area ratio of the two proton chemical shifts on C=C in the GMA moiety and the proton chemical shifts in the SCS ring [14,19]. The FTIR spectrum also designated the successful preparation of SCS-GMA (Figure 2b). The stretching vibration of C=O in the ester group was presented at 1650 cm^−1^. The absorption peak at 2926 cm^−1^ matches the stretching vibration of the asymmetric -CH_3_ and -CH_2_. The absorption peak at 3440 cm^−1^ was generated with the stretching vibrations of amino (-NH_3_) and hydroxyl (-OH) groups in the SCS molecule [15]. The ring-opening reaction of epoxy with the amino group is a highly efficient nucleophilic substitution [20]. The preparation process is simple and green, and the double bonds introduced on the chitosan chain provide sites for binding between molecular chains.

### 2.2. Preparation and Characterization of the SA/SCS Gel and PEI/PDRN Complexes

**Figure 3:**
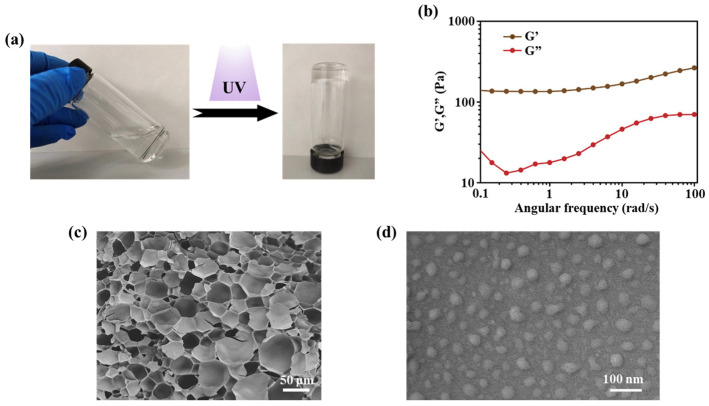
(**a**) Gelation progression of the SA/SCS gel. (**b**) Storage modulus (G′) and loss modulus (G″) of the SA/SCS gel. (**c**) SEM image of the SA/SCS gel. (**d**) TEM image of PEI/PDRN complexes at the N/P of 10.

The abundance of carboxyl groups suspended on the SA chain produce negative charges after ionization [21]. Therefore, when the prepared SCS-GMA is mixed with the SA, the SCS-GMA chains are entangled with the SA chains through physical entanglement and electrostatic interactions [22]. The non-covalent bond interaction between molecular chains can endow hydrogels with self-healing ability [23]. This will help the hydrogel closely adhere to the wound surface. In order to ensure structural stability and mechanical properties after gelation, the biocompatible photoinitiator lrgacure 2959 is used to initiate reactions between double bonds. Under UV irradiation for 2 min, lrgacure 2959 generates free radicals and activates the addition reaction between double bonds, thereby connecting the SCS-GMA chain together. The gelation process is shown in Figure 3a. The viscoelasticity of the SA/SCS gel involved rheology measurement. As illustrated in Figure 3b, the storage modulus (G′) is significantly above the loss modulus (G″), indicating the elastic dominant property of the SA/SCS gel. The mixture before gelation is injectable, allowing it to act on irregular wounds. This performance is due to the dynamic network structure of the hydrogel. After a short period of UV irradiation, with the creation of covalent bonds among molecular chains, a hydrogel dressing as a shield is formed. A typical 3D porous structure of the SA/SCS gel was observed using SEM (Figure 3c). This 3D network structure can enable the gel to absorb the wound exudate, sustain a moist environment, and allow gas exchange. As a nucleic acid delivery system, it needs to be able to effectively condense nucleic acid. PEI and DNA complexes with a N/P ratio of 10 could achieve a balance between transfection efficiency and toxicity [14]. The morphology of the PEI/PDRN complex revealed with TEM is shown in Figure 3d. Complexes smaller than 100 nm were observed. The nano-vector can be encased in the hydrogel, released at the wound site, and subsequently absorbed by cells with great efficiency.

### 2.3. In Vitro Biocompatibility

**Figure 4:**
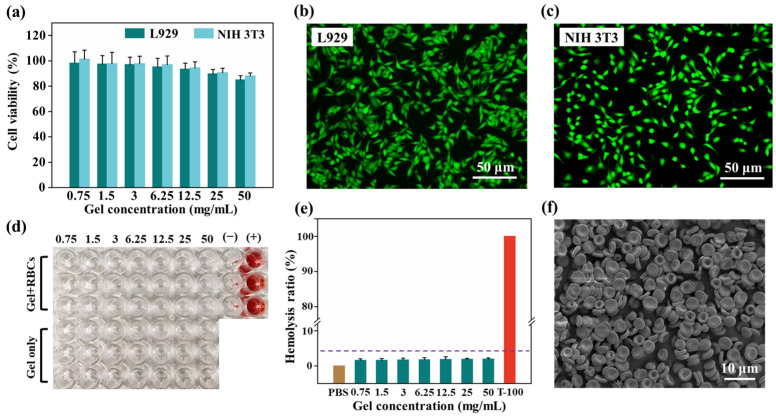
(**a**) Cell viability of the leachate from the SA/SCS gel in L929 and NIH 3T3 cell lines. The living/dead cell double staining of (**b**) L929 and (**c**) NIH 3T3 cells (Green: live cells; Red: dead cells). (**d**) Hemolysis assessment of the SA/SCS gel (mg/mL; −: negative control; +: positive control). (**e**) Hemolysis rate of the SA/SCS gel; mean ± s.d.; *n* = 3. (**f**) The SEM photo of RBCs treated with SA/SCS gel.

Cell viability and hemocompatibility are important indicators for evaluating the biocompatibility of materials. Natural polysaccharides possess good biological safety. As illustrated in Figure 4a, the cell viability of L929 and NIH 3T3 cells in different concentrations of leachate is higher than 80%. The live/dead staining experiment can also confirm that cells grew well in the leachate (Figure 4b,c). After co-incubation with the exudate, plenty of living cells stained with a green color were observed in both cell lines. There are almost no dead cells that should be dyed red. Figure 4d illustrates certainly that hemolysis was not observed within the erythrocyte suspension treated with SA/SCS gel, specifying the excellent hemocompatibility of the SA/SCS gel. Within the tested concentrations, all the hemolysis rates remained below 5% (Figure 4e). Visually observed with SEM (Figure 4f), RBCs remain intact when treated with SA/SCS gel. The preparation process of green chemistry and the use of safe raw materials are the guarantee of biosafety. The above results confirm that the prepared SA/SCS gel has good biocompatibility and can be further explored for its in vivo application.

### 2.4. PDRN Release Profile and Cell Proliferation

**Figure 5:**
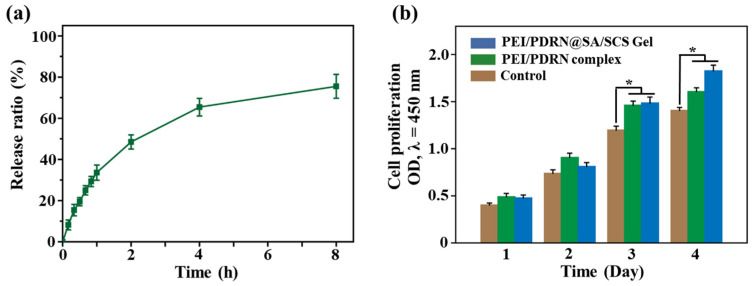
(**a**) Release profiles of the PEI/PDRN@SA/SCS gel. (**b**) Cell proliferation assay of L929 cells treated with PEI/PDRN complexes and PEI/PDRN@SA/SCS gel (mean ± SD, *n* = 3), * *p* < 0.05.

PDRN can interact with A_2A_ receptors, thus promoting tissue regeneration, angiogenesis, and an anti-inflammatory effect [24]. The dry gel was immersed in the PEI/PDRN complex solution, and complexes were encapsulated into the gel along with the solution adsorption. A sustained release, rather than a sudden release, was recorded (Figure 5a). This release profile will facilitate the long-term effect of PDRN at the wound site. As demonstrated in Figure 5b, the proliferation of L929 cells treated with PEI/PDRN complexes and PEI/PDRN@SA/SCS gel was accelerated. The PEI/PDRN-complex-treated group exhibited the highest proliferation rate on day 2. This may be due to the high concentration of PDRN in the PEI/PDRN-complex-treated group in the previous few days. As time went on, the slow release and accumulation of PDRN in the hydrogel made the effect of the PEI/PDRN@SA/SCS gel treatment group more durable, so it showed the highest proliferation-promoting effect from day 3. This sustained therapeutic effect may be beneficial for wound repair, especially for a curative of chronic wounds.

### 2.5. Antibacterial Ability

**Figure 6:**
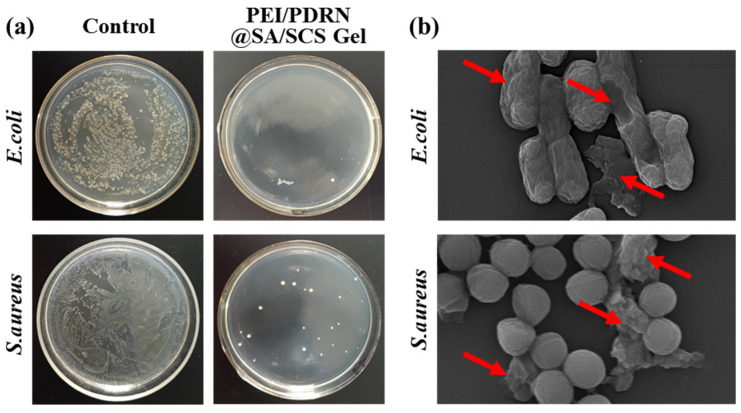
(**a**) Antibacterial testing of PEI/PDRN@SA/SCS gel against *E. coli* and *S. aureus*. (**b**) The SEM photos of bacteria treated with PEI/PDRN@SA/SCS gel (the red arrow indicates the ruptured outer membrane).

Bacteria can make wounds infected and difficult to heal [25]. Hydrogel dressings can shield bacteria and other harmful substances. In addition, the positive charge carried by the amino group on the SCS backbone in the matrix can break the bacterial cell membrane, thereby killing bacteria [26]. As shown in Figure 6a, the amount of *E. coli* and *S. aureus* colonies decreased significantly once being treated with the PEI/PDRN@SA/SCS gel. Microscopically, the bacterial structure changes under the action of polycations. SEM photos revealed that after PEI/PDRN@SA/SCS gel treatment, the bacterial outer membrane was damaged and there was content leakage (Figure 6b). The ideal antibacterial effect allows this hydrogel dressing to prevent bacterial infection during application. This performance is significant for infected wounds and chronic wounds. Note that, unlike antibacterial dressings that release antibiotics or metal ions, PEI/PDRN@SA/SCS gel utilizes the cation of chitosan to achieve the antibacterial activity, which can avoid the development of bacterial resistance.

### 2.6. In Vivo Wound Healing Efficacy

**Figure 7:**
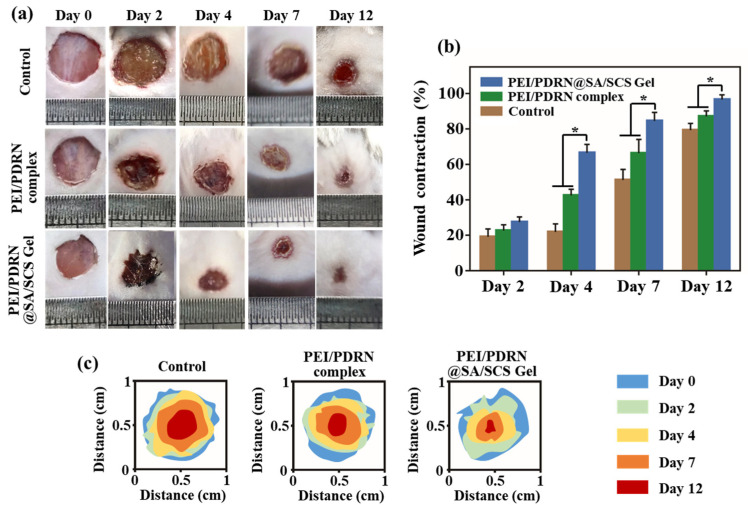
(**a**) Photos of the wound area on day 0, 2, 4, 7, and 12. (**b**) Wound healing rates at predetermined intervals, * *p* < 0.05. (**c**) Wound closure traces on day 0, 2, 4, 7, and 12.

A mouse full-thickness skin wound model was established to assess the influence of PEI/PDRN@SA/SCS gel on wound healing. The dressing was changed every 2 days. The camera recorded the gross observation of the wound (Figure 7a) and the wound contraction ratio was calculated (Figure 7b). All wounds showed healing with reduced wound area. The wounds of untreated mice (control group) healed slowly. In comparison, the PEI/PDRN complex group showed a certain healing-promoting effect. As predicted, the wound healing rate of the PEI/PDRN@SA/SCS gel group was faster than that of other groups. At the terminal point, the wounds cured with PEI/PDRN@SA/SCS gel were almost closed completely, while other groups still exhibited significant remaining wound areas. By computing the restoration area (Figure 7c), the PEI/PDRN@SA/SCS-gel-treated group showed the best healing-promoting effect with the wound closure ratio of 97% on the last day, while the closure ratio of the blank group and the PEI/PDRN-complex-treated group was about 80% and 88%, respectively. The wound treated with the PEI/PDRN complex exhibited the second-best therapeutic effect at the initial stage. This trend is consistent with the study of cell proliferation. Nano-vectors make it easier for nucleic acids to enter cells. This is because the positively charged PEI is easily adsorbed on the negatively charged cell membrane. Compared with naked PDRN, cells are more likely to uptake vectors with endocytosis [24]. In addition to the healing-promoting effect of PDRN in the hydrogel PEI/PDRN@SA/SCS gel, the hydrogel dressing provided a microenvironment and barrier function for wound healing. In conclusion, with the support of the nano-nucleic-acid-delivery system, PDRN-loaded polysaccharide-based hydrogel showed the best therapeutic effect.

## 3. Materials and Methods

### 3.1. Materials

Short-chain chitosan (SCS, degree of deacetylation > 90%) was obtained from Jinhu Crust Product Co., Ltd. Branched polyethylenimine (PEI, 25 kDa), lrgacure 2959, sodium alginate (SA), and glycidyl methacrylate (GMA) were purchased from Energy Chemical Co. (Shanghai, China). The polydeoxyribonucleotide (PDRN) was obtained from ReaLi Tide Biological Technology Co., Ltd. (Weihai, China). A living/dead-cell double-staining kit, cell counting kit-8 (CCK-8 kit), a PicoGreen dsDNA assay kit, thiazolyl blue tetrazolium bromide (MTT), and triton X-100 were obtained from Solarbio Science & Technology Ltd. Fetal bovine serum (FBS), Dulbecco’s Modified Eagle’s Medium (DMEM), penicillin–streptomycin, and trypsin were obtained from Gibco Life Technologies (Carlsbad, CA, USA). Escherichia coli (*E. coli*, ATCC 25922) and Staphylococcus aureus (*S. aureus*, ATCC 6538) were acquired from the American Type Culture Collection (ATCC).

### 3.2. Preparation of SCS-GMA

Briefly, 2.7 mL (20 mmol) of GMA was dropped into 18 mL of ethanol and mixed evenly. In total, 1 g of SCS was adequately dissolved in 100 mL of DI water. The SCS solution was then mixed with the GMA solution described above. The mixture was stirred at room temperature for 30 min and then stirred at 80 °C for an additional 4 h. The SCS-GMA was obtained with dialysis (MWCO 3500) and lyophilization. Nuclear magnetic resonance spectroscopy (NMR, Bruker DRX-400) and Fourier transform infrared (FTIR, Thermo Scientific, Waltham, MA, USA) were utilized to confirm the structure of the product.

### 3.3. Preparation of SA/SCS Gel and PEI/PDRN Complexes

The same mass of SA (50 mg) and SCS-GMA (50 mg) was dropped into 8 mL of DI water, and then 5 mg of lrgacure 2959 in 2 mL of DI water was supplemented. The solution was agitated evenly and then evacuated to remove excess bubbles. The hydrogel (termed as SA/SCS gel) was formed after 100 min of irradiation with a UV lamp. The morphology of the hydrogel was recorded with scanning electron microscopy (SEM, JSM-7800F) after freeze-drying. The dynamic rheology of the hydrogel was measured with a rotating rheometer (25 °C, 1–100 rad/s).

PEI/PDRN complexes were formed by mixing equal volumes of PEI and PDRN solutions at the N/P ratio of 10. Each mixture was vortexed and incubated for 30 min at room temperature. The complexes were observed with TEM (TEM, JEOL TEM-2100 Plus). Before observation, the complexes’ solution was applied to a copper grid and stained with a 0.2% (*w*/*v*) phosphotungstic acid aqueous solution.

### 3.4. Cell Viability Evaluation

The biocompatibility of the hydrogel was analyzed with the MTT assay [27]. The hydrogel leachates with a gradient concentration (immersed in the medium for 48 h) were collected. L929 and NIH 3T3 cells (10,000 cells/well) were planted in 96-well plates and cultivated for 24 h. The incubation was carried out according to the standard process. The medium was substituted with the leachate. After continuous cultivation for 24 h, the leachate was abandoned and 100 μL of MTT (0.5 mg/mL) was added to each well. The leachate was replaced with 100 μL of DMSO after 4 h. The absorbance values (at 490 nm) were recorded with a microplate reader (Bio-Rad model 680). The cell viability was calculated according to the subsequent formula: Cell viability (%) = [A]_test_ − [A]_0_)/[A]_control_ × 100%. [A]_test_ is the average absorbance value of the wells containing leachate, [A]_0_ is the average absorbance value of the wells containing sterile water, and [A]_control_ is the average absorbance value of the wells containing the medium. The average absorbance was calculated from six parallel wells.

The living/dead-cell double-staining experiment of L929 and NIH 3T3 cells was carried out. The cells were incubated in a 24-well plate (10,000 cells/well) and cultivated for 24 h. Then, the medium was replaced with the leachate (50 mg/mL). After 24 h, the cells were stained with the living/dead-cell double-staining kit according to the instructions and photographed via an inverted fluorescence microscope.

### 3.5. Hemolysis Test

The hemolysis test of the prepared hydrogel was executed as stated in reported processes [28]. Red blood cell (RBC) suspensions were mixed with an equal volume of gradient concentrations of SA/SCS gel, PBS, and triton X-100, respectively. The deposit was separated with centrifugation (2000 rpm, 10 min) after being incubated for 1 h at 37 °C. The UV absorbance (at 570 nm) of the supernatant was recorded. The hemolysis rate was calculated according to the subsequent formula: Hemolysis rate (%) = (A_test_ − A_neg_)/(A_pos_ − A_neg_) × 100%. A_test_ is the absorbance value of the supernatant containing samples, A_neg_ is the absorbance value of the supernatant containing PBS, and A_pos_ represents the absorbance value of the supernatant containing triton X-100. The morphology of the RBCs was photographed with SEM.

### 3.6. PDRN Release Profile

The lyophilized SA/SCS gel was soaked in 2 mL of a PEI/PDRN complex aqueous solution (N/P = 10, containing 120 μg of PDRN) to obtain the PDRN-loaded SA/SCS gel (termed as PEI/PDRN@SA/SCS gel). The PEI/PDRN@SA/SCS gel was dialyzed against 3 mL of a PBS buffer at 37 °C in a dialysis bag (MWCO 7000). At predetermined time intervals, 5 μL of PBS was collected. The amount of PDRN was calculated according to the operating instructions of the PicoGreen dsDNA assay kit.

### 3.7. Cell Proliferation Test

L929 cells were planted in a 96-well plate and incubated under typical conditions as described above for 24 h. Then, the medium was substituted with a refresh culture medium containing PEI/PDRN complexes (N/P = 10, containing 6 μg of PDRN) or PEI/PDRN@SA/SCS gel (1 mg/mL, containing 6 μg of PDRN). The optical density (450 nm) of the medium was recorded with the CCK-8 kit on days 1, 2, 3, and 4.

### 3.8. Antibacterial Activity Assay

Briefly, 5 mg of SA/SCS gel was spread in a 24-well plate. Place a small amount of sterile PBS nearby the sample to avoid water loss during the experiment. In total, 10 μL of the *E. coli* or *S. aureus* suspension (10^8^ CFU/mL) was smeared on the surface of the SA/SCS gel and incubated for 5 h. Subsequently, bacteria were rinsed from the hydrogel with 2 mL of sterile PBS. In total, 10 μL of a bacterial suspension was equably smeared on the agar plate. After being incubated for 12 h at 37 °C, the number of colonies on the plate was counted. In total, 10 μL of a bacterial suspension (10^8^ CFU/mL) was smeared on an agar plate as a control. The bacterial suspensions were washed with sterile PBS, fixed with glutaraldehyde (5%), and dehydrated with ethanol, successively. Then, the morphology of bacteria was photographed with SEM.

### 3.9. Wound Healing Study

Kunming mice (6–7 weeks old, male) were arbitrarily divided into 3 groups (5 rats in each group): non-treated control group (blank), PEI/PDRN complex group (1 mL, containing 60 μg of PDRN), and PEI/PDRN@SA/SCS gel group (containing 60 μg of PDRN). The mice were anesthetized with isoflurane and a full-thickness wound with a diameter of about 0.8 cm was created on their back. On days 3, 6, 9, and 12, the size of the wounds was recorded and the dressings were refreshed. The wound contraction rate was calculated according to the following formula: (Area _day 0_ − Area _day n_)/Area _day 0_ × 100%.

### 3.10. Statistical Analysis

The last values were expressed as the mean (±standard deviation (SD)). Student’s t test was used for statistical significance. *p* values were set to less than 0.05. All experiments were repeated at least three times.

## 4. Conclusions

A composite hydrogel for delivering PDRN was prepared. This wound healing strategy combined with nucleic-acid-based therapy showed an ideal effect. The double-bond-functionalized SCS has good water solubility and retains the antibacterial property of chitosan. Through the electrostatic interaction with SA and the photoinduced double-bond addition reaction, an antibacterial and healing-promoting hydrogel was constructed. The plasticity before UV curing makes the prepared dressing suitable for wounds with irregular shapes. The prepared hydrogels possess good biocompatibility. PEI and PDRN interact with each other through positive and negative charges to form nanoscale vectors. The nucleic acid delivery system avoids the premature degradation of PDRN and helps PDRN enter the cells at the wound site. The in vivo wound healing study reveals that the PDRN-loaded SA/SCS gel can effectively promote wound repair. The proposed hydrogel dressing is simple regarding preparation and may also have the potential for clinical transformation.

## Data Availability

The data presented in this study are available upon request from the corresponding author.
